# Supplementary material for: The rise of angiosperms strengthened fire feedbacks and improved the regulation of atmospheric oxygen
Source: Nat Commun. 2021 Jan 21;12:503. doi: 10.1038/s41467-020-20772-2 (PMC7820256; doi:10.1038/s41467-020-20772-2)
Supplement: Supplementary file 1 — Supplementary Information [file 41467_2020_20772_MOESM1_ESM.pdf]

Supplementary Information For:

The rise of angiosperms strengthened fire feedbacks and improved the regulation of atmospheric oxygen

Claire M. Belcher[1, 3*], Benjamin J. W. Mills[2], Rayanne Vitali[1, 3],
Sarah J. Baker[1, 3], Timothy M. Lenton[3], Andrew J. Watson[3].

[1]wildFIRE Lab, University of Exeter, Exeter EX4 4PS, UK
[2]School of Earth and Environment, University of Leeds, Leeds LS2 9TJ, UK
[3]Global System Institute, University of Exeter, Exeter EX4 4QE, UK
Contact c.belcher@exeter.ac.uk

Documentation for the COPSE biogeochemical model

Supplementary Note 1 - Background

COPSE is a global biogeochemical model, which computes the major long-term fluxes of carbon, oxygen, phosphorus, nitrogen and sulfur through the hydrosphere and crust. COPSE is a 'forwards' model in which processes are driven by a set of evolving boundary conditions (forcing factors) and internal dynamics, including a nutrient-driven biosphere. COPSE was first constructed by Bergman et al.,[1] who combined the GEOCARB model of[2] with a model for ocean nutrient cycling and productivity[3,4]. This is the most up-to-date version of the model, which is described and tested in[5], with further additions in[6]. This supplement provides all equations necessary to run the model, but for full details see the aforementioned papers.

Supplementary Note 2

The model uses a single box to represent the atmosphere and ocean, and boxes to represent the sedimentary inventories of the different chemical species. There are no spatial dimensions in the COPSE model.

Supplementary Table 1

Model species are shown in table 1 below. Each inventory is allowed to evolve during the model run.

Description	Name	Exists in box	Size at present
Atmospheric CO_2	A	Hydrosphere	3.193×10^{18} mol C
Buried organic C	G	Crust	1.25×10^{21} mol C
Buried carbonate C	C	Crust	5.0×10^{21} mol C
Ocean sulfate	S	Hydrosphere	4×10^{19} mol S
Buried pyrite sulfur	PYR	Crust	1.8×10^{20} mol S
Buried gypsum sulfur	GYP	Crust	2.0×10^{20} mol S
Ocean phosphate	P	Hydrosphere	3.1×10^{15} mol P
Ocean nitrate	N	Hydrosphere	4.35×10^{16} mol N
Atmospheric oxygen	O	Hydrosphere	3.7×10^{19} mol O

Supplementary Note 3 - Model Equations

The following equations dictate the inputs and outputs of each of the model reservoirs.

Marine phosphate: $\frac{dP}{dx} = psea - f_{mopb} - f_{capb} - f_{fepb}$

Atmospheric oxygen: $\frac{dO}{dx} = f_{locb} + f_{mocb} - f_{oxidw} - f_{ocdeg} + 2(f_{mpsb} - f_{pyrw} - f_{pyrdeg})$

Hydrosphere carbon: $\frac{dA}{dx} = f_{oxidw} + f_{carbw} + f_{ocdeg} + f_{ccdeg} - f_{locb} - f_{mocb} - f_{mccb} - f_{sfw}$

Marine sulfate: $\frac{dS}{dx} = f_{gypw} + f_{pyrw} + f_{gypdeg} + f_{pyrdeg} - f_{mpsb} - f_{mgsb}$

Buried organic carbon: $\frac{dG}{dx} = f_{locb} + f_{mocb} - f_{oxidw} - f_{ocdeg}$

Buried carbonate carbon: $\frac{dC}{dx} = f_{mccb} + f_{sfw} - f_{carbw} - f_{ccdeg}$

Buried pyrite S: $\frac{dPYR}{dx} = f_{mpsb} - f_{pyrw} - f_{pyrdeg}$

Buried gypsum S: $\frac{dGYP}{dx} = f_{mgsb} - f_{gypw} - f_{gypdeg}$

Marine nitrate: $\frac{dN}{dx} = f_{nfix} - f_{denit} - f_{monb}$

Model fluxes

Model fluxes are described below. They generally take the form of a present day rate multiplied by a series of scalings, which include the size of the parent reservoir, forcing factors, and non-flux calculations such as temperature or the degree of marine anoxia.

Degassing: sediment to hydrosphere

Carbonate C degassing: $f_{ccdeg} = k_{ccdeg} \cdot D \cdot \left(\frac{C}{C_0}\right)$

Organic C degassing: $f_{ccdeg} = k_{ocdeg} \cdot D \cdot B \cdot \left(\frac{G}{G_0}\right)$

Pyrite S degassing: $f_{pyrdeg} = k_{pyrdeg} \cdot D \cdot \left(\frac{PYR}{PYR_0}\right)$

Gypsum S degassing: $f_{gypdeg} = k_{gypdeg} \cdot D \cdot \left(\frac{GYP}{GYP_0}\right)$

Weathering: sediment to hydrosphere

Oxidative C weathering: $f_{oxidw} = k_{oxidw} \cdot U^{Usil} \cdot \left(\frac{G}{G_0}\right) \cdot \left(\frac{O}{O_0}\right)^{0.5}$

Carbonate C weathering: $f_{carbw} = k_{carbw} \cdot U^{Ucarb} \cdot \left(\frac{C}{C_0}\right) \cdot CA \cdot PG \cdot f_{biota} \cdot g_T$

Pyrite S weathering: $f_{pyrw} = k_{pyrw} \cdot U^{Usil} \cdot \left(\frac{PYR}{PYR_0}\right)$

Gypsum S weathering: $f_{gypw} = k_{gypw} \cdot \frac{f_{carbw}}{k_{carbw}} \cdot \left(\frac{GYP}{GYP_0}\right)$

Phosphorus weathering: $f_{phosw} = k_{phosw} \cdot EP \cdot \left\{ kp_{sil} \left(\frac{f_{silw}}{k_{silw}}\right) + kp_{carb} \left(\frac{f_{carbw}}{k_{carbw}}\right) + kp_{ox} \left(\frac{f_{oxidw}}{k_{oxidw}}\right) \right\}$

Burial: hydrosphere to sediment

Marine organic C burial: $f_{mocb} = k_{mocb} \cdot \left(\frac{newp}{newp_0}\right)^2$

Land organic C burial: $f_{locb} = k_{locb} \cdot \left(\frac{p_{land}}{p_{land_0}}\right) \cdot CP_{land}$

Marine carbonate burial: $f_{mccb} = f_{silw} + f_{carbw}$

Marine pyrite S burial: $f_{mpsb} = k_{mpsb} \cdot \left(\frac{S}{S_0}\right) \cdot \left(\frac{O_0}{O}\right) \cdot \left(\frac{f_{mocb}}{k_{mocb}}\right)$

Marine gypsum S burial: $f_{mgsb} = k_{mgsb} \cdot \left(\frac{S}{S_0}\right) \cdot CAL$

Fe-phosphate burial: $f_{fepb} = k_{fepb} \cdot \left(\frac{1-ANOX}{k_{oxfrac}}\right) \cdot \left(\frac{P}{P_0}\right)$

Ca-phosphate burial: $f_{capb} = k_{capb} \cdot \left(\frac{f_{mocb}}{k_{mocb}}\right)$

Organic P burial: $f_{mopb} = \left(\frac{f_{mocb}}{CP_{sea}}\right)$

Organic N burial: $f_{monb} = \left(\frac{f_{mocb}}{CN_{sea}}\right)$

Internal fluxes:

Granite weathering: $f_{granw} = k_{granw} \cdot U^{kw_{sil}} \cdot GA \cdot PG \cdot f_{biota} \cdot f_{T_{gran}}$

Basalt weathering: $f_{basw} = k_{basw} \cdot BA \cdot PG \cdot f_{biota} \cdot f_{T_{bas}}$

Silicate weathering: $f_{silw} = f_{granw} + f_{basw}$

Denitrification: $f_{denit} = k_{denit} \cdot \left(1 + \left(\frac{ANOX}{1-k_{oxfrac}}\right)\right) \cdot \left(\frac{N}{N_0}\right)$

Nitrogen fixation: $f_{nfix} = k_{nfix} \cdot \left(\frac{P-\frac{N}{16}}{P_0-\frac{N_0}{16}}\right)^2$

Marine new production: $newp = 117 \cdot \min\left(\frac{[N]}{16}, [P]\right)$

P flux to land: $p_{land} = k_{landfrac} \cdot VEG \cdot f_{phosw}$

P flux to sea: $p_{sea} = f_{phosw} - p_{land}$

Non-flux calculations

Carbon atmospheric fraction $atfrac = atfrac_0 \cdot \left(\frac{A}{A_0}\right)$

Relative atmospheric CO_2: $RCO_2 = (\frac{A}{A_0}) \cdot \left(\frac{atfrac}{atfrac_0}\right)$

Atmospheric O_2 mixing ratio: $O_{2_{mr}} = \frac{\frac{O}{O_0}}{\frac{O}{O_0}+k_{mr}}$

Global average surface temperature: $T_{gast} = 15 + climsens \cdot \frac{\log RCO_2}{\log(2)} - k_l \cdot \left(\frac{t}{570}\right)$

Average temperature for weathering: $T_{surf} = T_{gast} \cdot k_{Tgradm} + k_{Tgradc}$

Granite weathering T effect: $f_{T_{gran}} = e^{0.0724(T_{surf}-15)} \cdot \left(1 + 0.038 \cdot (T_{surf} - 15)\right)^{0.65}$

Basalt weathering T effect: $f_{T_{bas}} = e^{0.0608(T_{surf}-15)} \cdot \left(1 + 0.038 \cdot (T_{surf} - 15)\right)^{0.65}$

Carbonate weathering T effect: $g_T = 1 + 0.087(T_{surf} - 15)$

Seafloor weathering T effect: $f_{T_{sfw}} = e^{0.0608(T_{surf}-15)}$

Temperature effect on vegetation:	$V_T = 1 - \left(\frac{T_{surf} - 25}{25}\right)^2$
CO_2 effect on vegetation:	$V_{CO_2} = \frac{CO_2 ppm - p_{minim}}{p_{half} + p_{atm} - p_{minim}}$
Oxygen effect on vegetation:	$V_{O_2} = 1.5 - 0.5\left(\frac{O}{O_0}\right)$
Overall limitation of terrestrial NPP:	$V_{NPP} = 2 \cdot EVO \cdot V_T \cdot V_{CO_2} \cdot V_{O_2}$
Fire ignition probability scaling:	$ignit = \min\left(max\left(48 \cdot O_{2_{mr}} - 9.08\,,\ 0\right)\right)$
Fire effect on terrestrial biomass:	$firef = \frac{k_{fire}}{k_{fire} - 1}$
Mass of terrestrial biota:	$VEG = V_{NPP} \cdot firef$
Terrestrial biota weathering effect:	$f_{biota} = \{1 - \min(V \cdot W, 1)\} \cdot k_{plantenhance} \cdot RCO_2^{0.5} + V \cdot W$
Marine P concentration:	$[P] = 2.2\left(\frac{P}{P_0}\right)$
Marine N concentration:	$[N] = 30.9\left(\frac{N}{N_0}\right)$
Marine anoxic fraction:	$ANOX = \frac{1}{1 + e^{-k_{anox} \cdot \left(k_u\left(\frac{newp}{newp_0}\right) - \left(\frac{O}{O_0}\right)\right)}}$

Supplementary Table 2

All model forcing factors are detailed below. All have the value of 1 at the present day and are nondimensional.

Description	Name	Based on
Tectonic degassing	D	Reconstructed subduction zone and rift lengths
Continental uplift	U	Sediment abundance
Carbonate burial depth	B	Fossil record
Basalt silicate exposed area	BA	Degassing and flood basalt emplacements
Granite silicate exposed area	GA	Paleogeographic reconstruction
Land plant evolution	EVO	Fossil record
Land plant weathering enhancement	W	Experimental and field studies
Land plant C:P ratio	CP_{land}	Sedimentary coal deposition record
Selective phosphorus weathering	EP	Experimental studies
Paleogeographic effect on weathering	PG	Climate modelling
Ocean calcium concentration	CAL	Fluid inclusion measurements

Supplementary Table 3 - Fixed parameters are shown in the table below. *except for k_{fire}

Description	Name	Value
Present day marine organic carbon burial	k_{mocb}	4.5×10^{12} mol C yr^{-1}
Present day land organic carbon burial	k_{locb}	4.5×10^{12} mol C yr^{-1}
Present day organic carbon degassing	k_{ogdeg}	1.25×10^{12} mol C yr^{-1}
Present day organic carbon weathering	k_{oxidw}	7.75×10^{12} mol C yr^{-1}
Present day carbonate burial	k_{mccb}	2.125×10^{13} mol C yr^{-1}
Present day carbonate degassing	k_{ccdeg}	1.5×10^{13} mol C yr^{-1}
Present day carbonate weathering	k_{carbw}	8×10^{12} mol C yr^{-1}
Present day seafloor weathering	k_{sfw}	1.75×10^{12} mol C yr^{-1}
Present day basalt weathering	k_{basw}	3.975×10^{12} mol C yr^{-1}
Present day silicate weathering	k_{sil}	1.325×10^{13} mol C yr^{-1}
Present day phosphorus weathering	k_{phosw}	5.1×10^{10} mol P yr^{-1}
Present day pyrite burial	k_{mpsb}	7×10^{11} mol S yr^{-1}
Present day gypsum burial	k_{mgsb}	2.5×10^{12} mol S yr^{-1}
Present day pyrite weathering	k_{pyrw}	4.5×10^{11} mol S yr^{-1}
Present day gypsum weathering	k_{gypw}	2×10^{12} mol S yr^{-1}
Present day pyrite degassing	k_{pyrdeg}	2.5×10^{11} mol S yr^{-1}
Present day gypsum degassing	k_{gypdeg}	5×10^{11} mol S yr^{-1}
Present day Ca-P burial	k_{capb}	2×10^{10} mol P yr^{-1}
Present day Fe-P burial	k_{fepb}	1×10^{10} mol P yr^{-1}
Present day nitrogen fixation	k_{nfix}	8.67×10^{12} mol N yr^{-1}
Present day denitrification	k_{denit}	4.3×10^{12} mol N yr^{-1}
Present day ocean oxic fraction	k_{oxfrac}	0.9975
Atmospheric O_2 mixing ratio conversion	k_{mr}	3.762
Pre-plant weathering enhancement factor	$k_{preplant}$	0.25
Uplift effect on carbonate weathering	kw_{carb}	0.9
Uplift effect on silicate weathering	kw_{sil}	0.33
Phosphorus input from silicate weathering	kp_{sil}	0.8
Phosphorus input from carbonate weathering	kp_{carb}	0.14
Phosphorus input from organic carbon oxidation	kp_{ox}	0.06
Fraction of phosphorus buried on land	$k_{landfrac}$	0.0588
C:P ratio of buried marine organics	CP_{sea}	250
C:N ratio of buried marine organics	CN_{sea}	37.5
Present day atmospheric fraction of CO_2	$atfrac_0$	0.01614
Long-term climate sensitivity	$climsens$	5 K
Solar luminosity difference at 570 Ma	k_l	7.4 W m^{-2}
Latitudinal temperature gradient slope	k_{Tgradm}	0.66
Latitudinal temperature gradient constant	k_{Tgradc}	4.95
Vegetation CO_2 minimum	$p_{minimum}$	10 ppm
Vegetation CO_2 half saturation	p_{half}	183.6 ppm
*Fire effect on vegetation biomass	k_{fire}	$evolving$
Steepness of anoxia transition	k_{anox}	12
Marine oxygen utilization parameter	k_u	0.5

Supplementary Figure 1

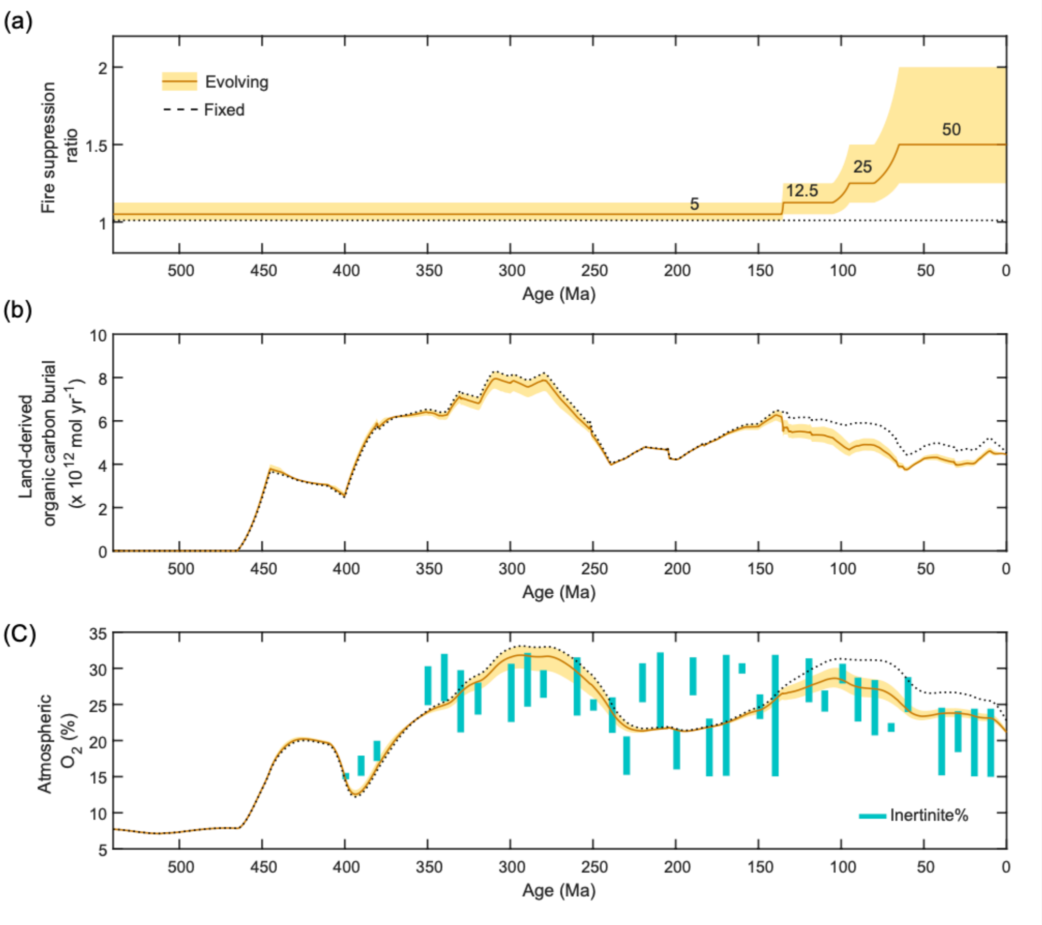

Supplementary Figure 1 Full Phanerozoic COPSE model run including the effect of the angiosperm evolving fire suppression implemented between 150 Ma to the present day. This suppression ratio is only relevant to the 50Ma as other mechanisms, that we have not yet researched, may come into effect after 50Ma. A. Yellow shaded area shows our evolving 'fire suppression ratio', representative of the changing fuel source due to angiosperm evolution. Black dashed line shows fixed weak fire feedback in the baseline COPSE model. B. Modelled land-derived organic carbon burial. C. Modelled atmospheric oxygen mixing ratio compared to inertinite-derived estimates from[7]

Supplementary References

1. Bergman, N.M., Lenton, T.M. & Watson, A.J. COPSE: A new model of biogeochemical cycling over Phanerozoic time. *Am. J. Sci.,* **304**, 397-437 (2004).

2. Berner, R.A. A model for atmospheric CO2 over Phanerozoic time. *Am. J. Sci.* **291**, 339-376 (1991).

3. Van Cappellen, P., Ingall, E.D. Redox stabilisation of the atmosphere and oceans by phosphorus-limited marine productivity. *Science*, **271**, 493-496 (1996).

4. Lenton, T.M. & Watson, A.J. Redfield revisited 2. What regulates the oxygen content of the atmosphere? *Global Biogeochemical Cycles*, B, **14**, 249-268 (2000).

5. Lenton T.M., Daines, S.J., Mills, B.J.W. COPSE reloaded: An improved model of biogeochemical cycling over Phanerozoic time. *Earth Sci. Rev.* **178**, 1-28 (2018).

6. Mills, B.J.W. Krause, A.J., Scotese, C.R., Hill, D.J., Shields, G.A., Lenton, T.M. Modeliing the long-term carbon cycle, atmospheric CO2, and Earth surface temperature from late Neoproterozoic to present day. *Gond. Res.,* **67**, 172-186 (2019).

7. Glasspool, I.J., Scott, A.C. Phanerozoic concentrations of atmospheric oxygen reconstructed from sedimentary charcoal. *Nat. Geosci*., **3**, 627-630 (2010).
