# Supplementary material for: The rise of angiosperms strengthened fire feedbacks and improved the regulation of atmospheric oxygen
Source: Nat Commun. 2021 Jan 21;12:503. doi: 10.1038/s41467-020-20772-2 (PMC7820256; doi:10.1038/s41467-020-20772-2)
Supplement: Supplementary file 3 — Description of Additional Supplementary Files [file 41467_2020_20772_MOESM3_ESM.pdf]

Description of Additional Supplementary Files

File Name: Supplementary Data 1
Description: COPSE model code and associated files
